# Mitogen Activated Protein Kinase (MPK) Interacts With Auxin Influx Carrier (OsAux/LAX1) Involved in Auxin Signaling in Plant

**DOI:** 10.1186/s12575-015-0025-7

**Published:** 2015-10-30

**Authors:** Tapan Kumar Mohanta, Nibedita Mohanta, Pratap Parida, Hanhong Bae

**Affiliations:** School of Biotechnology, Yeungnam University, Gyeongsan, 712749 Republic of Korea; Department of Biotechnology, North Orissa University, Sri Ramhandra Vihar, Takatpur, Orissa 757003 India; Regional Medical Research Center, NE Region, Indian Council of Medical Research Dibrugarh, 786001 Assam, India

**Keywords:** OsAux/LAX, Mitogen activated protein kinase (MPK), Phosphorylation, Yeast two-hybrid

## Abstract

**Background:**

Mitogen activated protein kinases (MPKs) are serine/threonine protein kinases that contain characteristic T-x-Y motif in the activation loop region. MPKs are important signaling molecules involved in diverse signaling cascades that regulate plant growth, development and stress responses by conducting phosphorylation events in their target proteins. MPKs phosphorylate their target proteins at either S-P/T-P (Serine/Proline/Threonine) amino acid. To understand, if MPKs are involved in the auxin signaling cascade, we identified probable target proteins of MPKs involved in auxin signaling or transport processes.

**Results:**

A genome-wide search of the rice genome database led us to identification of the OsAux/LAX1 gene as a potential downstream target protein of MPKs. In-silico analysis predicted that MPKs interact with OsAux/LAX1 proteins which were validated by a yeast two-hybrid assay that showed OsMPK3, OsMPK4 and OsMPK6 are physically interact with OsAux/LAX1 protein.

**Conclusion:**

The yeast two-hybrid interaction showed that MPKs are directly involved in auxin signaling events in plants. This is the first study to report direct involvement of MPKs in the auxin signaling pathway.

## Background

The plant mitogen activated protein kinases (MPKs) are evolutionarily conserved serine/threonine protein kinases that contain a characteristic T-x-Y motif in the activation loop region and group specific conserved docking domains in the C-terminal region [1, 2]. MPKs are involved in highly conserved signal transduction cascade that consists of at least three kinase modules. The kinase module contains a MP3K (mitogen activated protein kinase kinase kinase), a MP2K (mitogen activated protein kinase kinase) and a MPK [2]. In the event of any environmental or cellular signaling process, plasma membrane activates MAP3Ks, which conserved serine/threonine protein kinases that phosphorylate downstream amino acids at the S/T-X_3–5_-S/T motif of MP2K in the activation loop domain. The MP2Ks then phosphorylate the downstream MPKs at the threonine and tyrosine residue of T-x-Y motif [1]. Once, MPKs are phosphorylated, they can able to phosphorylate a wide array of downstream substrate proteins including other kinases, proteins and transcription factors to regulate gene expression [1, 3, 4]. The integrity of phosphorylation events of specific MPK with their substrate proteins is mediated by shared docking domains and adaptor proteins [1, 5].

The plant MPKs pathway is a major and well developed pathway involved in growth, development and biotic and abiotic stress responses in plants [2, 6]. This pathway is very complex and involves crosstalk with several other pathways [6–8]; therefore, the present study was conducted to decipher the complex interaction mechanism involved in plant MPKs and their involvement in auxin signaling events. Sorensson et al., [9] reported that, MPKs phosphorylates their downstream target proteins either at S-P-R/S-S-P-R/S-P-K/S-S-P-K consensus sequences [9]. Therefore, we investigated whether; OsAux/LAX1 is a suitable interacting partner of MPK as it contains the S-P motif at position 88. Therefore, we planned to conduct interaction analysis of rice OsMPKs and OsAux/LAX1 protein to confirm their physical interaction. An in-silico interaction study (docking interaction) was conducted to determine the details of the interacting amino acids of OsMPK and OsAux/LAX1 proteins (docking and protein-protein interaction). The results obtained by the in-silico interaction study were validated by a yeast two hybrid interaction assay. This is the first study to explain the direct involvement of the MPK pathway in auxin signaling events.

## Results and Discussion

### Sequence Retrieval, Template Identification, Homology Modeling and Structural Analysis

**Fig. 1:**
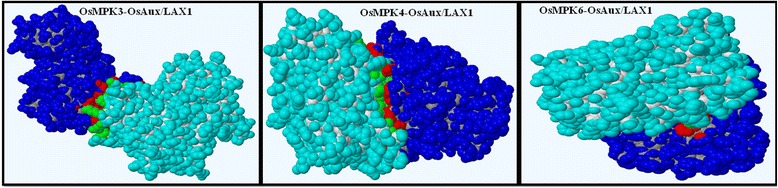
Protein-protein docking orientations of the homology models of OsMPK and OsAux/LAX after building the dimers. OsMPKs are shown in blue and OsAux/LAX1 protein is shown in cyan. The interacting residues of OsMPKs are shown in red color whereas the interacting residues of OsAux/LAX1 are shown in green

**Fig. 2:**
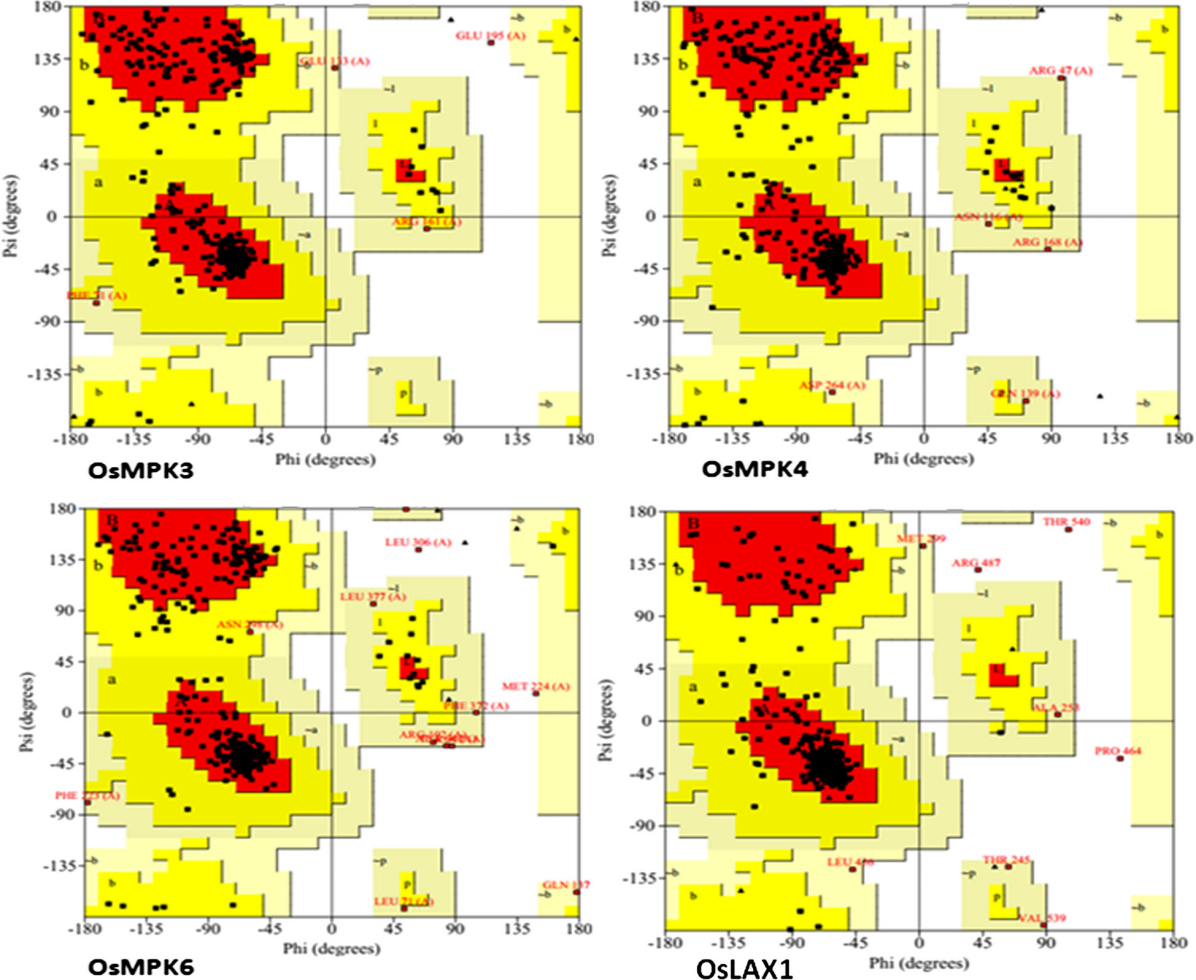
Ramachandran plot analysis of the homology models of OsMPKs and OsAux/LAX. Plot statistics for OsAux/LAX1: Residues in most favored regions [A, B, L] 305 (90.2 %), Residues in additional allowed regions [a, b, l, p] 26 (7.7 %), Residues in generously allowed regions [~a, ~b, ~l, ~p] 5 (1.5 %), Residues in disallowed regions 2 (0.6 %), Number of non-glycine and non-proline residues 338 (100.0 %). Number of end-residues (excl. Gly and Pro) 2, Number of glycine residues (shown as triangles) 30, Number of proline residues 15, Total number of residues are 385; OsMPK3: Residues in most favored regions [A,B,L] 269 (87.3 %), residues in additional allowed regions [a, b, l, p] 35 (11.4 %), residues in generously allowed regions [~a, ~b, ~l, ~p] 3 (1.0 %), residues in disallowed regions 1 (0.3 %). Number of non-glycine and non-proline residues 308 (100.0 %). Number of end-residues (excluding Gly and Pro) 1, number of glycine residues (shown as triangles) 11, number of proline residues 22, Total number of residues are 342; OsMPK4: Residues in most favored regions [A, B, L] 270 (85.7 %), residues in additional allowed regions [a, b, l, p] 40 (12.7 %), residues in generously allowed regions [~a, ~b, ~l, ~p] 5 (1.6 %), residues in disallowed regions 0 (0.0 %). Number of non-glycine and non-proline residues 315 (100.0 %). Number of end-residues (excl. Gly and Pro) 0, number of glycine residues (shown as triangles) 16, number of proline residues 21, Total number of residues are 352; OsMPK6: Residues in most favored regions [A, B, L] 249 (82.5 %), residues in additional allowed regions [a, b, l, p] 41 (13.6 %), residues in generously allowed regions [~a, ~b, ~l, ~p] 10 (3.3 %), residues in disallowed regions 2 (0.7 %). Number of non-glycine and non-proline residues are 302 (100.0 %). Number of end-residues (excl. Gly and Pro) 1, number of glycine residues (shown as triangles) 11, number of proline residues 19, total number of residues are 333

The FASTA format amino acid sequences of OsMPK3, OsMPK4, and OsMPK6 were subjected to BLAST (basic local alignment search tool) and LOMETS server [10] to reveal the best templates for comparative modeling of both the proteins. Homology models were built based on the structure of the templates (Fig. 1). The homology models were analyzed for a broad study of the proteins. The modeled structures were validated by performing full geometric analysis with Procheck [11]. The structures were also analyzed with Modeval [12] (Table 1) which calculates and analyzes the main chain bond lengths, bond angles, stereochemistry of main and side chains, Ramachandran plots, and G factors, which in turn reflects the quality of the prediction. In Procheck, a low G-factor indicates that the property corresponds to a low-probability conformation and residues falling in the disallowed region of Ramachandran plot will have a low G-factor. It observes the steriochemical distribution of steriochemical parameters like torsion angles (phi-si combination, chi1-chi2 combination, chi-1 torsion for residues that don’t have chi-2, combined chi-3 and chi-4 torsion angles and omega torsion angles) and covalent geometry (main-chain bond length and main-chain bond angle). Ramachandran plot is known to be the most reliable method of determining the quality of a modeled protein structure [13, 14]. ProCheck results revealed that more than 85 % of the residues of the models were present in the favored region, whereas less than 1 % of the amino acids were present in the forbidden part of the Ramachandran plot (Fig. 2, Table 1). ERRAT was used to identify non-bonded interactions statistics amid different types of atoms [15] and the overall quality was found to be more than 80 % (Table 1). As shown in Table 1, VERIFY3D passed the congeniality of the three dimensional atomic model with its own amino acid sequences [16, 17]. The results of structural super position revealed a very low root mean square deviation (RMSD) between target and template structures indicating their high structural similarity.

**Table 1:**
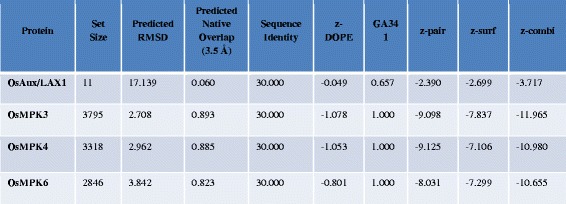
Procheck Analysis of OsMPKs and OsAux/LAX1

z-score of protein access the quality of model using the normalized DOPE (Discrete Optimized Potential Energy) method. The DOPE is based on an improved reference state that corresponds to noninteracting atoms in a homogenous sphere with the radius dependent on sample native structure and thus it counts for the finite and spherical shape of the native structures. A positive Z-score are likely to be poor models, while the scores lower than −1 or so are likely to be good acceptable model. **GA341**: GA341 parameter derived from the statistical potential and shows the reliability of a protein model. A model is predicted to be most reliable when the model score is higher than pre-specified cutoff (0.7) and has probability of the correct fold that is larger than 95 %. A protein model is considered correct when the C-alpha atom superpose within 3.5A^o^ of their correct position. **z-pair**: A pairwise statistical potential that contributes to GA341. **z-surf**: a surface statistical potential that contributes to GA341. **z-combi**: a combined statistical potential that contributes to GA341

### Protein-protein Docking Studies

**Fig. 3:**
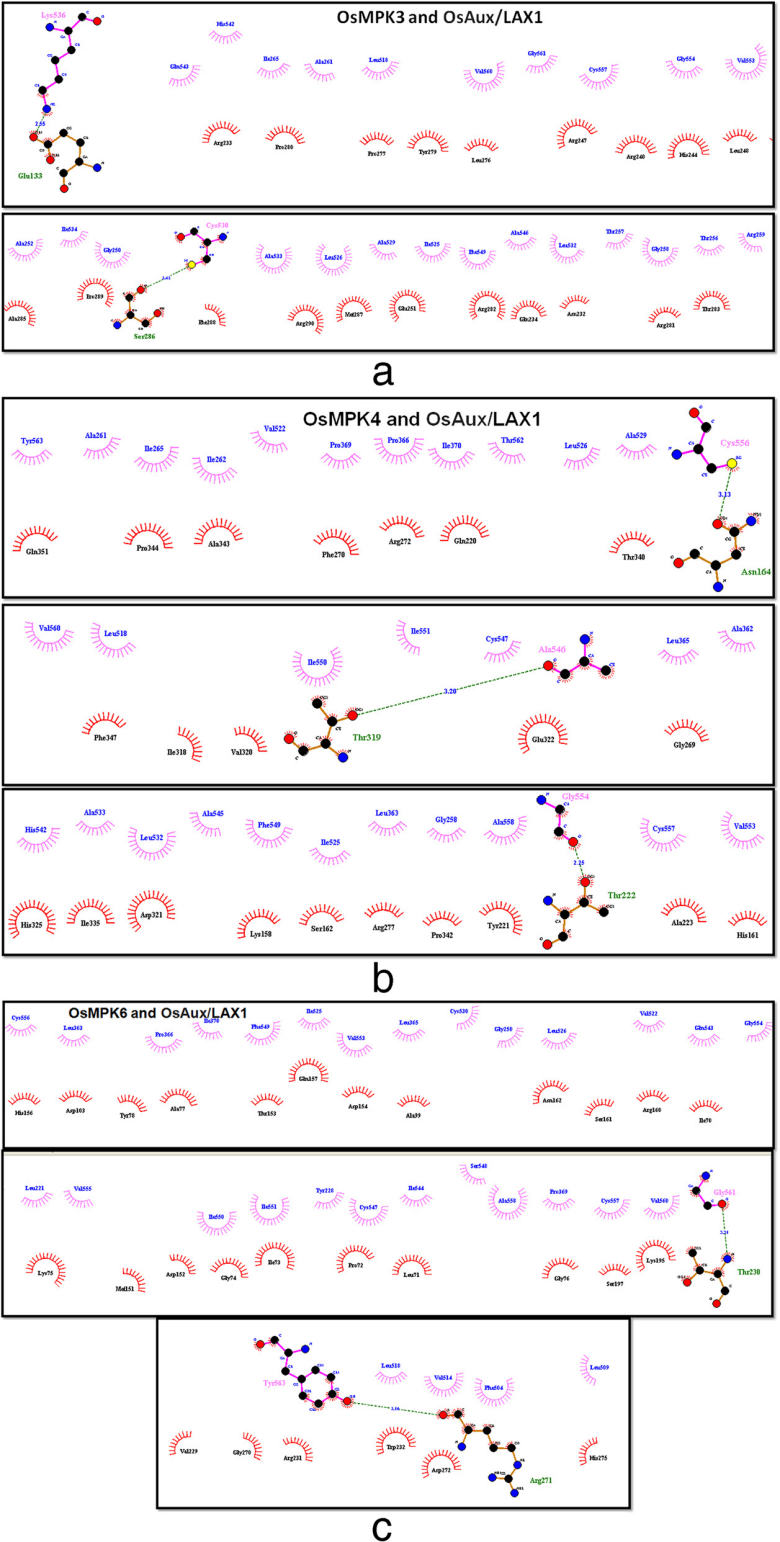
Hydrophobic and hydrogen bond forming residues of OsMPK and OsAux/LAX1. **a** Hydrophobic and hydrogen bond forming residues of OsMPK3-LAX1 dimer. OsMPK3 residues are shown at the top in blue and LAX1 residues shown at the bottom in black. Hydrophobic interactions are shown without any line and hydrogen bonds are shown as green dotted lines. **b** Hydrophobic and hydrogen bond forming residues of OsMPK4-OsAux/LAX1 dimer. OsMPK4 residues are shown at the top in blue and LAX1 residues are shown at the bottom in black. Hydrophobic interactions are shown without any lines and hydrogen bonds are shown as green dotted lines. **c** Hydrophobic and hydrogen bond forming residues of OsMPK6-OsAux/LAX1 dimer. OsMPK6 residues are shown at the top in blue and LAX1 residues are shown at the bottom in black. Hydrophobic interactions are shown without any lines and hydrogen bonds are shown as green dotted lines

The OsMPK proteins were docked with OsAux/LAX1 using the GRAMM-X docking server [18]. Rigid body docking was performed and the orientation was checked. The OsMPK protein was taken as the receptor, whereas the OsAux/LAX1 protein was considered as ligand (Fig. 1). The initial orientation of the docked complex from GRAMM-X was refined using the RosettaDock server, which performs a local docking search. The server requires a desirable starting position to place the protein interfaces residues in position to interact with each other. The local perturbation of the RosettaDock server was ~ ±3A^o^ in the direction between the receptor and ligand, ~8A^o^ for the sliding of the surfaces, ~8^o^ of tilt, and 360^o^ spin around the axis at the centers of the target proteins. A total of 1000 simulations were performed using the server and ten best scoring complexes were selected for a detailed study based on the lowest energy. The Yasara server was used to conduct an energy minimization simulation study [19]. Dimers obtained from the Rosetta server were further submitted to the Yasara server for energy minimization using a GROMOS96 force field [19]. The dimer energy was initially very high. The docked complexes were minimized to the lowest scores as well and the lowest minimization energy (Fig. 3).

### Protein-protein Interaction Analysis

After conducting the necessary minimization steps, the protein complexes were submitted to Dimplot to identify the interaction sites (Fig. 3) [20]. Additionally, the hydrogen and hydrophobic bonds formed by the OsMPK-OsAux/LAX1 complexes were analyzed using Pymol (Fig. 3), while Dimplot was used to analyze the dimers and plot the various hydrogen and hydrophobic interactions. Amino acids Lys536 and Cys530 of OsMPK3 form hydrogen bonds with Glu33 and Ser286 of OsAux/LAX respectively at distances of 2.55 and 2.42 A^o^ (Fig. 3a). Amino acids Cys556, Ala546 and Gly554 of OsMPK4 form hydrogen bonds with Asn164, Thr319, and Thr222 respectively, at distances of OsAux/LAX1 with distance 3.13, 3.20 and 2.25 A^o^ (Fig. 3b). Amino acids Gly561 and Tyr563 of OsMPK6 form hydrogen bonds with Thr230 and Arg271 of OsAux/LAX1 at distances of 3.31 and 3.16 A^o^ (Fig. 3c).

### Yeast Two-hybrid Interactions of MPKs and LAX1

Auxin is an important hormone that regulates growth, development, tropism, apical dominance and several other processes in plants [21–23] as well as plays a crucial role in root development [6, 24, 25]. Auxin is synthesized in the aerial parts of the plant and transported toward the root tip to facilitate root development [26]. The transport of auxin from the aerial part of the plant to the root tip is conducted by specialized auxin transporter molecules, popularly known as auxin influx and efflux carriers [27–32] in a polarized manner [33]. Auxin influx carrier (Aux/LAX), a transmembrane amino acid transporter infuses the auxin molecule into the cell and the efflux carrier exports the auxin molecule to the adjacent cell in polarized manner [34–36]. Transport of the auxin molecule across the plasma membrane is an active process; therefore, the carrier molecules must be activated for the process to occur [35, 37]. Protein phosphorylation by kinase is one of the most important process that phosphorylates the target protein and leads to activation so it can carry out its active process [1, 38]. Mitogen activated protein kinases are most important family proteins found in plants which enable diverse cellular processes [1, 2]. Mitogen activated protein kinases have been reported to phosphorylate the target protein at the serine/proline (SP) or threonine/proline (TP) amino acid (S/T-P motif) [9]. We found that the auxin signaling protein, OsAux/LAX1 contains an S-P motif at 88^th^ position indicating that MPKs might phosphorylates the OsAux/LAX1 protein. To carry out phosphorylation events in the target protein (OsAux/LAX1), OsMPK first interacts with the protein via a hydrogen bond (Fig. 3) after which it carries out its phosphorylation event. The protein-protein interaction sites are different from the phosphorylation sites.

**Fig. 4:**
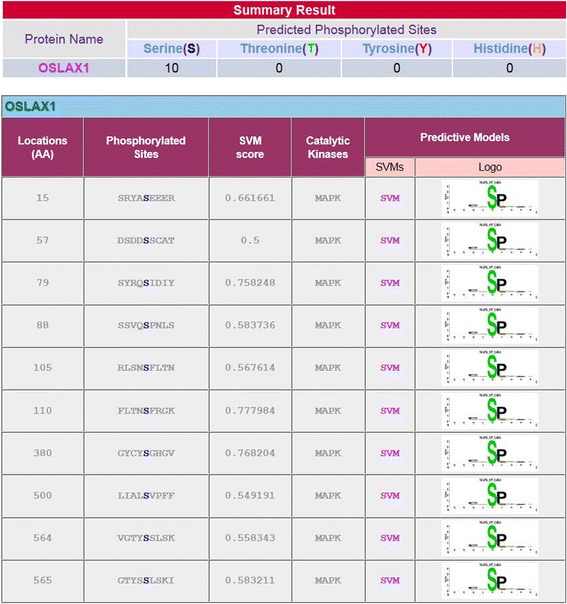
Phosphorylation site of OsAux/LAX1 predicted to be phosphorylated by MPKs. The prediction was conducted using Kinasephos2.0 server (http://kinasephos2.mbc.nctu.edu.tw/). The OsAux/LAX1 protein sequence was utilized to identify putative phosphorylation sites of MPKs

**Fig. 5:**
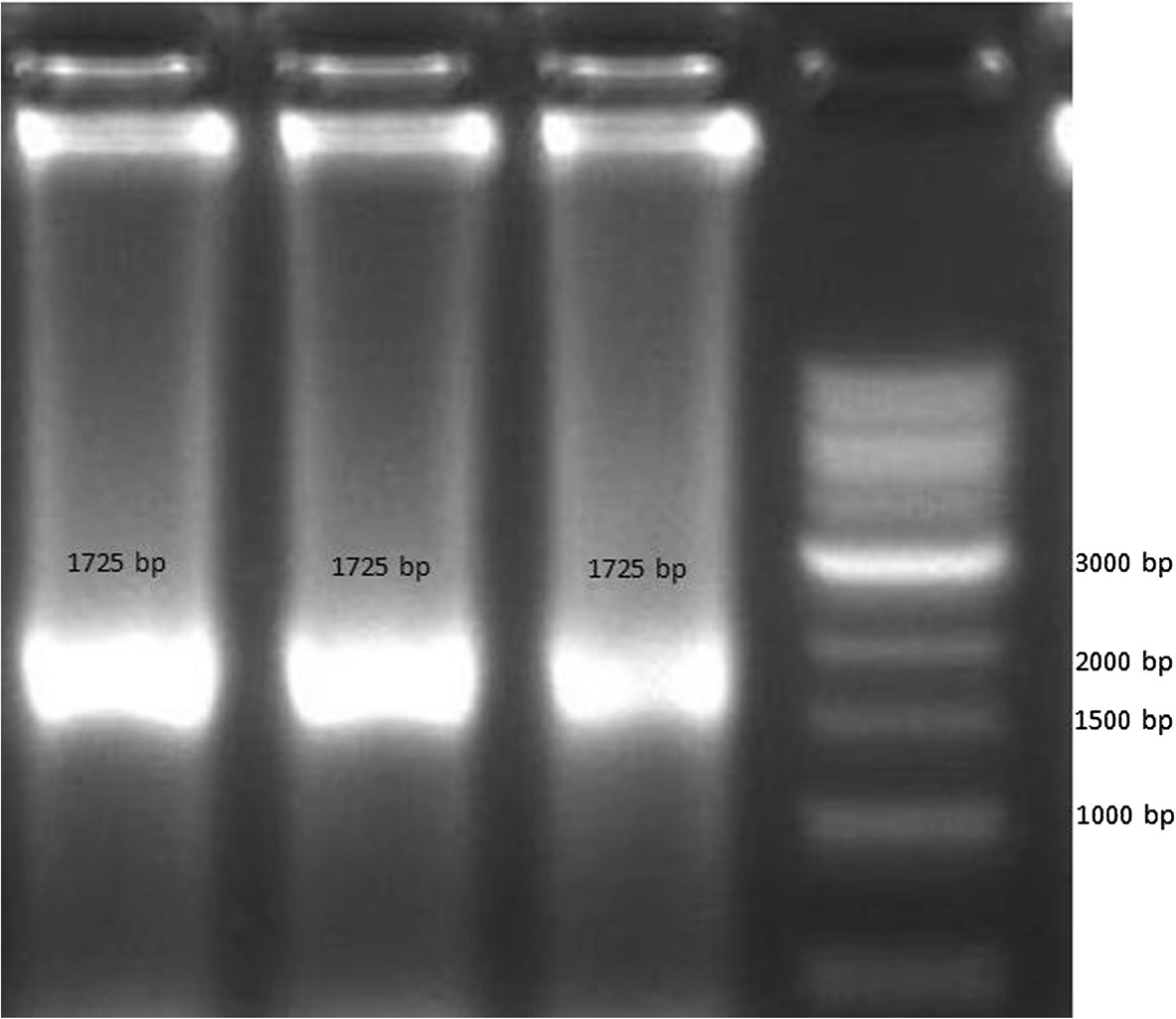
Agarose gel electrophoresis photograph of cloned OsAux/LAX1 gene. The amplified gene is 1725 nucleotides long

**Fig. 6:**
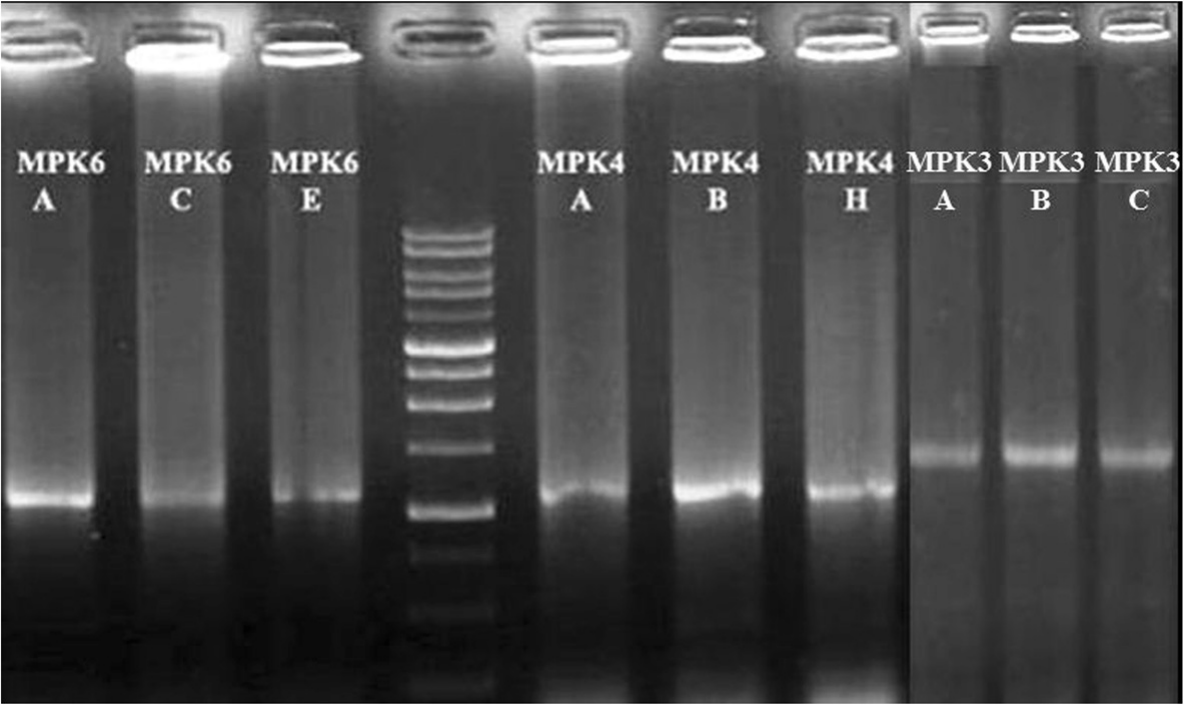
Agarose gel electrophoresis photograph of cloned OsMPKs. Amplified OsMPK3, OsMPK4 and OsMPK6 genes are 1110, 1131 and 1197 nucleotides long, respectively. The letter **a, b, c** (OsMPK3); **a, b, h** (OsMPK4); **a, c, e** (OsMPK6) in gel photograph of colony PCR of MPKs represents different selection plate names from where transformed colonies were taken to run colony PCR

**Fig. 7:**
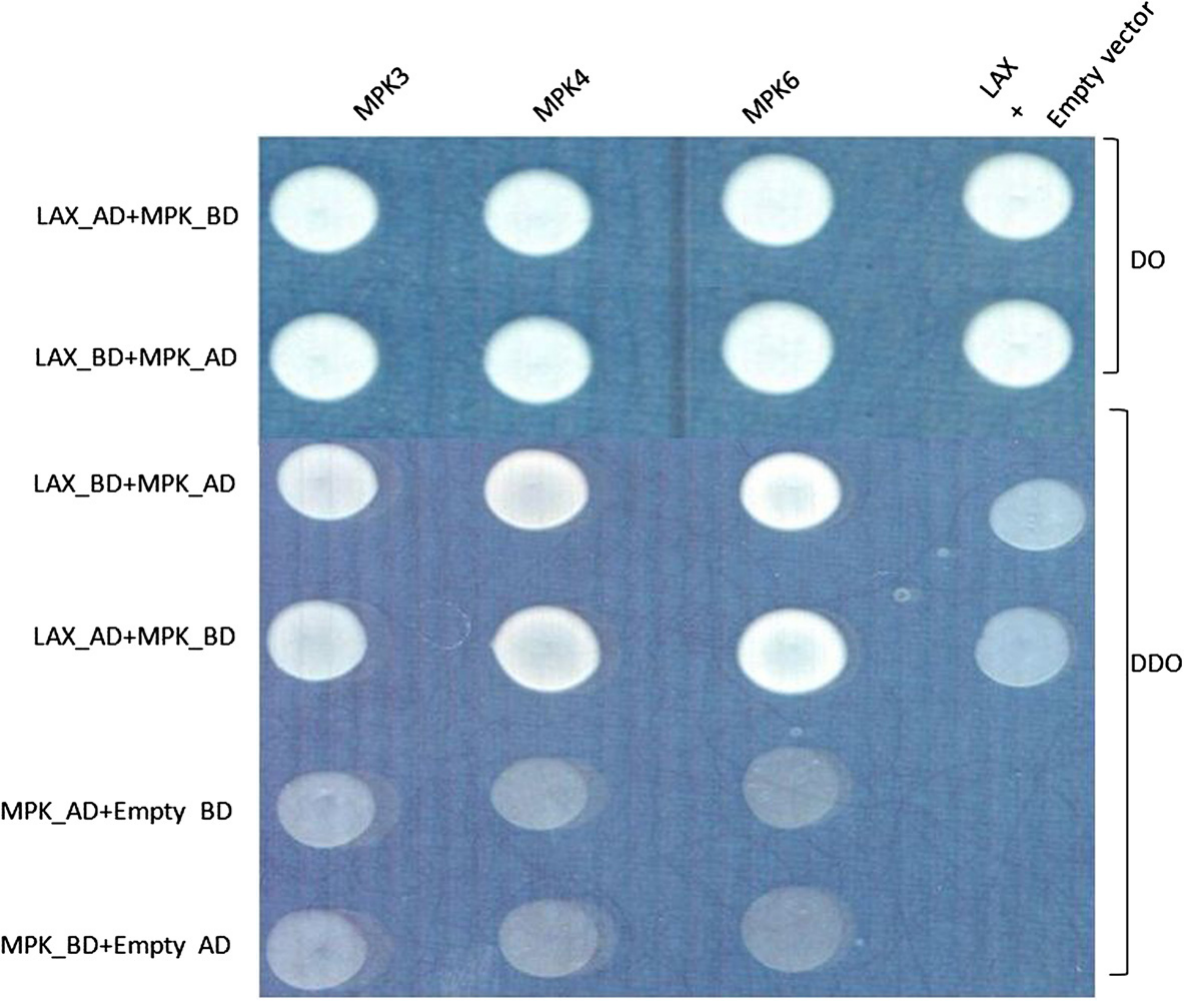
Yeast two-hybrid interaction of assay of OsMPKs and OsAux/LAX1. The transformed yeast constructs were grown in selection media [drop out (DO) and double drop out (DDO)]. Drop out media lacks -Leu/-Trp and double drop out media lacks –Ade/- His/-Leu and –Trp amino acids. In the study, the OsAux/LAX1 gene was incorporated into BD vector and OsMPK genes were incorporated into AD vector and vice versa. A swapping assay was conducted by incorporating OsAux/LAX1 into AD vector and OsMPKs in BD vector. Transformed colonies were first grown in DO media. The colonies raised in DO were then plated in DDO media. The colony those grown in DDO media were considered to be interacting with each other. OsAux/LAX1 in AD vector and OsMPKs in BD vector and vice versa grew in DDO media, confirming that OsAux/LAX1 interacts with MPKs. When OsAux/LAX1 gene in AD and BD vector was transformed with empty vector (BD and AD, respectively), colonies were observed in DO media but not in DDO media. Similarly, when OsMPKs in AD and BD vector were transformed with empty BD and empty AD vector, no colonies were observed in DDO media. This confirms that neither OsAux/LAX1 nor OsMPKs were able to grow in DDO media due to lack of their interacting gene. Absence of colonies in DDO media in empty vector confirms that, there is no auto-activation of yeast-two hybrid assay and the interactions are positive

To reconfirm the presence of potential MPK phosphorylation sites in OsAux/LAX1, we conducted in-silico prediction to identify phosphorylation of the OsAux/LAX1 protein using the kinasephos2.0 server [39]. OsAux/LAX1 was found to contain at least ten putative potential phosphorylation sites that could be phosphorylated by MPKs (Fig. 4). Although it OsAux/LAX1 was predicted to have ten potential MPK phosphorylation sites, it contained an S-P motif at 88^th^ position, indicating that this location was most likely to undergo phosphorylation by MPK. Therefore, we cloned the OsAux/LAX1 (Fig. 5) and OsMPKs (OsMPK3, OsMPK4 and OsMPK6) (Fig. 6) genes with suitable restriction sites (SmaI and NcoI) (Table 2). Transformation was conducted by inserting the OsAux/LAX1 gene into the AD vector and the OsMPKs gene into the BD vector. Transformed yeast constructs were then grown in selection media [(DO) drop out and (DDO) double drop out]. The yeast-two hybrid result in drop out (DO) media that lack of -Leu/-Trp amino acids shows, OsMPK3, OsMPK4 and OsMPK6 interacts with OsLAX1 and even colony was developed OsLAX1 transformed with empty vector (AD and BD) (Fig. 7) [40]. The empty vector did not contain any construct of the OsMPK gene. To reconfirm these findings, the colonies obtained from DO media were again sub-cultured in double drop out (DDO) media that lacking the -Ade/-His/-Leu and–Trp (Fig. 7) amino acids [40]. These results suggest that, OsMPK3, OsMPK4 and OsMPK6 interact physically with OsAux/LAX1 (Fig. 7). As shown in the figure, OsAux/LAX1 in AD vector transformed with OsMPKs in BD vector, resulted in development of colony in DDO media. Similarly, colonies were observed when OsAux/LAX1 in BD vector was transformed with OsMPK construct present in AD vector (swapping experiment), colony was observed (Fig. 7). Taken together, these finding indicate that OsMPKs and Aux/LAX1 interacted with each other. When OsMPKs constructs were transformed with either empty AD or empty BD vector in double drop out (DDO) media, no colonies were developed (Fig. 7) suggesting that, OsMPKs and OsAux/LAX1 protein physically interact with each other and did not grow in DDO media due to absence of interacting partner genes. These finding indicates that OsMPKs and OsAux/LAX1 interact physically with each other.

**Table 2:**
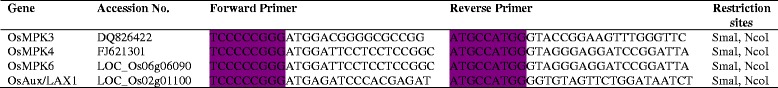
Forward and Reverse Primer Sequences used to Clone the OsMPKs and OsAux/LAX1 Genes

The highlighted portion indicates the restriction sites added with the primer sequence

## Conclusion

Auxin signaling event is crucial to growth and development of plants. However, the auxin signaling pathway is complex and involves interactions with several cascades. The result of the present study indicated that the MPK cascade is involved in auxin signaling events. This is the first report regarding involvement of MPK pathway in auxin signaling events.

## Methods

### Sequence Retrieval and Homology Modeling

Prior to homology modeling, the sequences of OsMPK3, and OsMPK4 were retrieved from the NCBI protein sequence database (http://www.ncbi.nlm.nih.gov/protein) in FASTA format. The sequences of OsMPK6 and OsAux/LAX1 were retrieved from the “rice genome annotation database” [41]. The Genebank accession numbers of OsMPK3, and OsMPK4 are DQ826422 and FJ621301 respectively while protein identification numbers of OsMPK6 and OsAux/LAX1 are LOC_Os06g06090 and LOC_Os02g01100 respectively (rice genome annotation project). An excellent relationship to study the protein primary and secondary structure can be achieved by homology based modeling [42]. It is possible to understand the protein function by computational modeling of a target protein using its proper template. This comparative modeling is based on the assumption that two proteins will have tertiary structure that shares a high percentage of similarity [42].

**Fig. 8:**
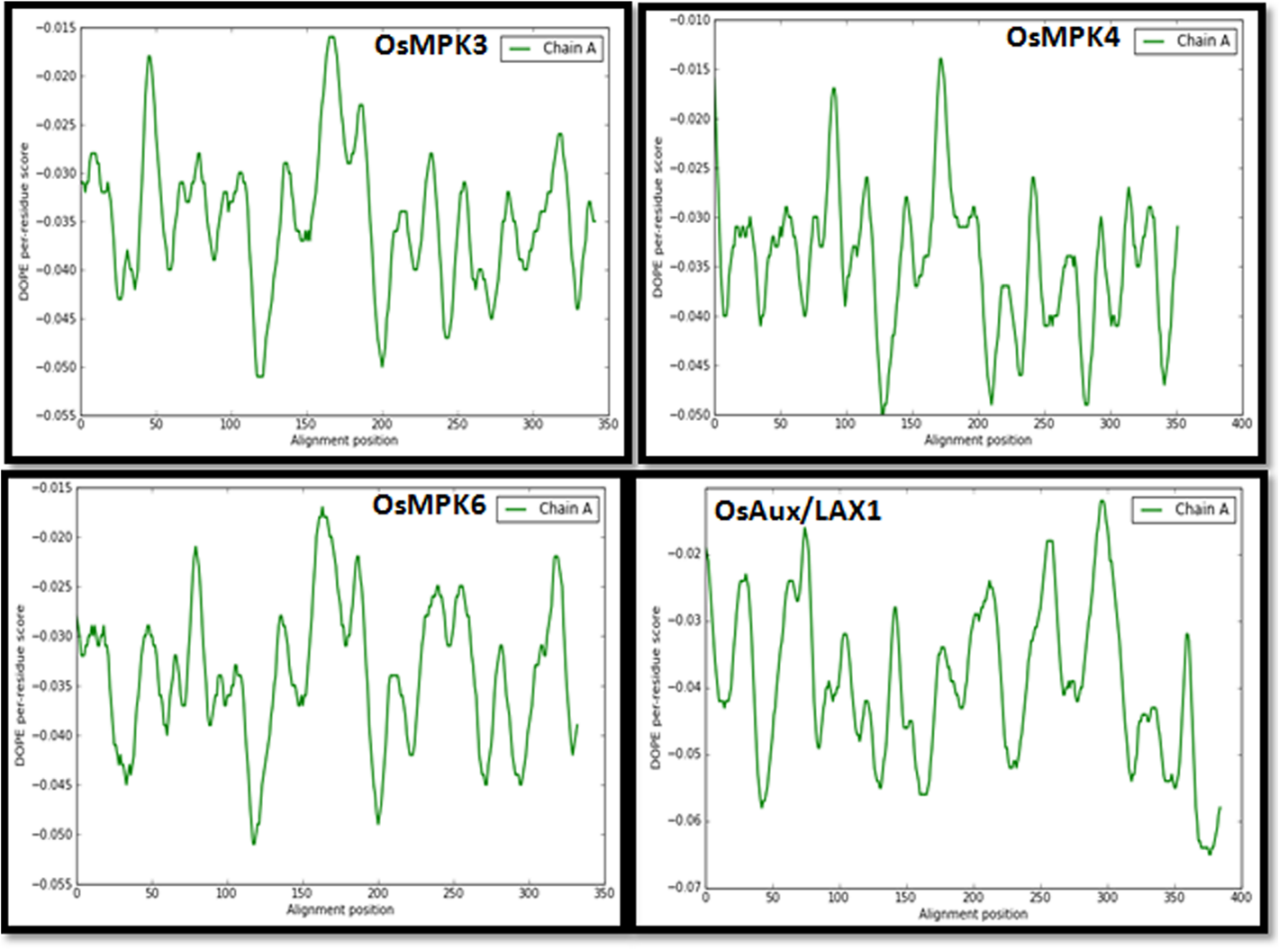
DOPE plot of OsMPKs and OsAux/LAX1. The protein residue numbers are plotted on the x-axis and the DOPE energies of each residue are plotted in the Y-axis. The predicted models of has lower optimized energy. The lower optimized energy confirms the higher stability of predicted protein model

Modeling was conducted using Modeller 9v11. Initially, 100 models were developed for the protein, from which only the model with lowest discrete optimized protein energy (DOPE) score was selected for further analysis (Fig. 8) [43]. A positive z- score in DOPE are likely to be poor models, while the scores lower than −1 or so are acceptable model. A model is predicted to be most reliable when the model score is higher than pre-specified cutoff (0.7) and has probability of the correct fold that is larger than 95 %. The target model was later refined by side chain refinement and loop modeling to increase the communion score of each residue. The loop prediction algorithms, LOOPER [44] and ChiRotor [45], were used to conduct the loop modeling and side chain refinement respectively.

### Structural Assessment

The models quality was checked by energetic and geometric means. The modeled homology structures were further validated using PROCHECK [11, 46] ERRAT [15] and VERIFY3D [16]. The PROCHECK software analyzes the stereochemical properties to assess quality of the Ramachandran plot, planarity of the peptide bond, the main chain hydrogen bond energy, Cα chiralities, non bonded interactions, and the overall G factor [46]. The ERRAT algorithm based on the statistical parameters of non-bonded interactions between different types of atoms and subsequently provides the accuracy of the protein model [15]. VERIFY3D checks the compatibility of the atomic models with its own amino acid sequence. A high VERIFY3D profile score indicates a better quality of model [16]. For further evaluation of the models, the ModEval Model Evaluation Server was used to calculate different model scores including z-Dope, GA341, z-pair, z-surf, and z-combi [12, 47, 48].

Protein-protein plays important roles in different biological processes, including signal transduction, gene expression, cellular transport, inhibition of enzyme activities, and the association of multi-domain proteins which leads to creation of stable protein-protein complexes important to meet their biological functions [49–51]. A protein-protein docking study was conducted to analyze the interaction of OsMPK3, OsMPK4 and OsMPK6 with OsAux/LAX1. The modeled structures were submitted to the GRAMM-X docking server [18] one at a time to achieve solid body docking using the fast Fourier transformation process by employing the smoothed Lennard-Jones potential, refinement stage and knowledge-based scoring, which provides the best surface match. Three dimers were formed after each successful docking, OsMPK3-OsAux/LAX1, OsMPK4-OsAux/LAX1 and OsMPK-OsAux/LAX1. The best dimer orientation found upon protein-protein docking, was again fed to the GRAMM-X server to obtain initial dimer orientations.

### Cloning of OsMPK and OsLAX1

The OsMPKs (OsMPK3, OsMPK4 and OsMPK6) sequences were cloned from *Oryza sativa* using the proper adapter primer sequences for restriction digestion, after which the full length cDNA was amplified (Table 2) [40]. Amplified OsMPKs and OsAux/LAX genes were confirmed by sequencing and all the clones were confirmed to be in the proper reading frame. The OsMPKs (OsMPK1, OsMPK2, OsMPK3) and OsAux/LAX1 genes were then cloned in pGADT7 and pGBKT7 vectors (BD Bioscience, USA) for yeast two-hybrid (Y2H) analysis as previously reported [40]. A match maker yeast two-hybrid assay kit was used to check the protein-protein interactions (BD Bioscience, USA).

Yeast competent cells (AH109) were prepared according to the manufacturer’s instruction for transformation of GADT7 and pGBKT7 vectors (BD Bioscience, USA). The OsMPKs and OsAux/LAX1 constructs were co-transformed for yeast two-hybrid analysis. Transformation was carried out in PEG/LiAc (polyethylene glycol/lithium acetate) solution at 30 °C for one hour in a water bath while shaking at 200 rpm. Transformed cells were then centrifuged at 700 x g for five minutes, after which the pellet was recovered and co-transformed constructs were plated in selected drop out (DO) nutrient medium that lacks -Leu and -Trp (SDO/-Leu/-Trp) amino acid. The colonies obtained from DO media were then streaked on selective double drop out (DDO) media deficient in the amino acids Ade, His, Leu and Trp (SD/-Ade/-His/-Leu/-Trp). Blank pGADT7 and pGBKT7 vectors were used as controls in both selective media.

## Abbreviations

MPK: Mitogen Activated Protein Kinase
MP2K: Mitogen Activated Protein Kinase Kinase
MP3K: Mitogen Activated Protein Kinase Kinase Kinase
OsAux/LAX: *Oryza sativa* Auxin Influx Carrier
DOPE: Discrete Optimized Protein Energy
Y2H: Yeast two Hybrid
SDO: Single Dropout
DDO: Double Dropout
